# Double adenomas of the pituitary reveal distinct lineage markers, copy number alterations, and epigenetic profiles

**DOI:** 10.1007/s11102-021-01164-1

**Published:** 2021-09-03

**Authors:** Christian Hagel, Ulrich Schüller, Jörg Flitsch, Ulrich J. Knappe, Udo Kellner, Markus Bergmann, Rolf Buslei, Michael Buchfelder, Thomas Rüdiger, Jochen Herms, Wolfgang Saeger

**Affiliations:** 1 Institute of Neuropathology, University Medical Center Hamburg-Eppendorf, Martinistr. 52, 20246 Hamburg, Germany; 2 Department of Pediatric Hematology and Oncology, University Medical Center Hamburg-Eppendorf, Martinistr. 52, 20246 Hamburg, Germany; 3 Children’s Cancer Research Center Hamburg, Martinistr. 52, 20251 Hamburg, Germany; 4 Department of Neurosurgery, University Medical Center Hamburg-Eppendorf, Martinistr. 52, 20246 Hamburg, Germany; 5 Department of Neurosurgery, University Hospital of the Ruhr-University Bochum, Hans-Nolte-Str. 1, 32427 Minden, Germany; 6 Institute of Pathology, Cytology & Molecular Pathology, Johannes-Wesling-Klinikum, University Hospital of the Ruhr-University Bochum, Hans-Nolte-Str. 1, 32427 Minden, Germany; 7 Institute of Neuropathology, Klinikum Bremen-Mitte, St Jürgen- Str. 1, 28205 Bremen, Germany; 8 Institute of Pathology, Sozialstiftung Bamberg, Buger Str. 80, 96049 Bamberg, Germany; 9 Neurochirurgische Klinik, Schwabachanlage 6, 91054 Erlangen, Germany; 10 Institute of Pathology, Städtisches Klinikum Karlsruhe gGmbH, Moltkestr. 90, 76133 Karlsruhe, Germany; 11 Center for Neuropathology and Prion Research, Ludwig-Maximilians-University Munich, Feodor-Lynen-Str. 23, 81377 Munich, Germany; 12 German Center for Neurodegenerative Diseases, Feodor-Lynen-Str. 17, 81377 Munich, Germany

**Keywords:** Pituitary, Double adenoma, Methylome profiling, Chromosomal gains and losses

## Abstract

**Purpose:**

Pituitary adenoma (PA) constitutes the third most common intracranial neoplasm. The mostly benign endocrine lesions express no hormone (null cell PA) or the pituitary hormone(s) of the cell lineage of origin. In 0.5–1.5% of surgical specimens and in up to 10% of autopsy cases, two or three seemingly separate PA may coincide. These multiple adenomas may express different hormones, but whether or not expression of lineage-restricted transcription factors and molecular features are distinct within multiple lesions remains unknown.

**Methods:**

Searching the data bank of the German Pituitary Tumor Registry 12 double pituitary adenomas with diverse lineage were identified among 3654 adenomas and 6 hypophyseal carcinomas diagnosed between 2012 and 2020. The double adenomas were investigated immunohistochemically for expression of hormones and lineage markers. In addition, chromosomal gains and losses as well as global DNA methylation profiles were assessed, whenever sufficient material was available (n = 8 PA).

**Results:**

In accordance with the literature, combinations of GH/prolactin/TSH–FSH/LH adenoma (4/12), GH/prolactin/TSH–ACTH adenoma (3/12), and ACTH–FSH/LH adenoma (3/12) were observed. Further, two out of 12 cases showed a combination of a GH/prolactin/TSH adenoma with a null-cell adenoma. Different expression pattern of hormones were confirmed by different expression of transcription factors in 11/12 patients. Finally, multiple lesions that were molecularly analysed in 4 patients displayed distinct copy number changes and global methylation pattern.

**Conclusion:**

Our data confirm and extend the knowledge on multiple PA and suggest that such lesions may origin from distinct cell types.

## Introduction

Pituitary adenomas typically comprise monoclonal proliferations of anterior pituitary cells. They are usually considered benign, although they may show invasive growth into neighbouring structures. Very rarely, they present as aggressive metastasising tumours referred to as pituitary carcinomas. PA constitute the third most common intracranial neoplasm after gliomas and meningiomas, representing approximately 15% of tumours in this localisation [1]. Adenoma cells either express no hormone (null cell PA) or the pituitary hormone(s) of the cell lineage of origin determined by specific transcription factors. In addition, about 1% of PA show a plurihormonal expression pattern [2], which may either be attributed to one cell lineage with aberrant expression of an additional hormone (monomorphous plurihormonal PA, one transcription factor), or rarely to two or three different cell lineages (plurimorphous plurihormonal PA, two or three transcription factors) [3]. In distinction to plurihormonal PAs, development of two or three separate PA may be observed in 0.5–1.5% of surgical specimens and in up to 10% of autopsy cases. These lesions are termed double or triple/multiple PAs [4, 5].

Genetic and epigenetic profiling of monomorphous PAs, which may help understanding the tumorigenesis and indicate possible therapeutic approaches, have been performed in several studies. The data disclosed a spectrum of alterations. Firstly, certain hereditary syndromes are associated with an increased incidence of PA (multiple endocrine neoplasia type 1: *MEN1*, Carney complex: *PRKAR1A*, familial isolated pituitary adenomas: *CDKN1B*, succinate dehydrogenases related paraganglioma syndrome: *SDH*s genes). Secondly, somatic *GNAS1* mutations were found in up to 40% of growth hormone (GH) expressing adenomas and *USP8* mutations in ACTH adenomas [6–8]. Thirdly, whole-exome sequencing revealed chromosomal losses in 25% of PAs, whereas only 2% showed chromosomal gains [7]. However, 60% of PA do not demonstrate genetic changes [9].

Epigenetic alterations in PA as determined by DNA methylation analysis revealed more hypomethylated regions in GH PA than in ACTH expressing PA. On the functional level, a hypomethylation of promoter regions of somatostatin receptor 5 (*SSTR5*), and growth hormone 1 and 2 (*GH1*, *GH2*) was found in GH PA compared to hypomethylation of proopiomelanocortin (*POMC*) in ACTH PA [7]. Other epigenetic changes in PA include histone modifications and altered expression of microRNAs (miRNA), which regulate mRNA activity [6]. However, the significance of these alterations for tumourigenesis, growth and hormone expression in PA is still obscure.

Concerning the classification of PA, application of mathematical algorithms to whole genome methylation data and transcriptome sequencing data revealed new aspects and relations between the different PA subgroups. Two recent studies [8, 10] were able to distinguish secreting corticotroph adenomas from silent (macro) corticotroph adenomas, the former harbouring *USP8* mutations and the latter showing an *USP8* wild type. In both studies gonadotroph adenomas showed a spatial relationship with silent ACTH PA in T-distributed Stochastic Neighbour Embedding (*t*-SNE) plots. Further, analysis of the transcriptome data presented by Neou et al. [8] grouped the WHO ‘‘null-cell subtype’’ PA together with gonadotroph tumours and the mixed GH-PRL PA with GH PA, separate from lactotroph tumours. Sparsely granulated GH PA clustered with thyreotroph and plurihormonal PIT1-positive PA, and not with the densely granulated GH PA as would be expected.

Molecular investigation of double PAs as tumour entities sharing the same general genetic and epigenetic background may possibly disclose similarities and differences in tumorigenesis and/or tumour differentiation. For this purpose the adenomas need to differ in their hormone profile; it does not suffice that the tumours appear as separate masses in MRI imaging [11]. In the present study we report 12 double PAs (DPA) with diverse hormone profile collected in the German Registry of Pituitary Tumours between 2012 and 2020, which were investigated in regard to their immunohistochemical expression pattern, and in 4 cases additionally concerning their chromosomal gains and losses and DNA methylation profile.

## Material and methods

### Tissue specimens

The data bank of the German Pituitary Tumor Registry was searched for double pituitary adenomas diagnosed between 2012 and 2020. Twelve cases were identified among 3654 surgical specimens of adenoma and 6 specimens of hypophyseal carcinoma accounting for 0.3% of all cases. Since immunohistochemical detection of transcription factors had been introduced in routine diagnostics only in the last 4 years, we had to rely on hormone expression pattern for identification of double adenomas. The German Pituitary Tumor Registry is a national reference centre that receives specimens from neurosurgeries throughout Germany and from pathologists for consulting examinations under the patronage of the German Society of Endocrinology [12].

### Histopathological and immunhistochemical investigation

Four routine assessment, the FFPE samples had been cut into 2 μm thick sections and were stained with H&E and PAS. In addition, the tissue had been investigated immunohistochemically for expression of pituitary hormones GH, prolactin (PRL), TSH, ACTH, FSH, LH, for alpha-subunit, for keratin Cam5.2 and the proliferation marker Ki67. For the present study, all cases were additionally labelled with antibodies against transcription factors Pit-1, T-pit, and SF-1 (Table 1).

**Table 1:** Clinical, histological, and molecular findings in 12 double Pas

Case	Sex	Age	Macro-adenoma	Invasive growth	Endocrinology	Tumour	Antigen-profile	Pit-1	T-pit	SF-1	Histological diagnosis	DNA methylation-based classifications (score)	Copy number variations
01	f	78	0	1	Cushing	A	ACTH++, Ki 1–2 %	0	1	0	Densely granulated ACTH-PA	Lack of DNA	n.a.
						B	LH+, Ki 1–2 %	0	0	0	FSH/LH-PA	Lack of DNA	n.a.
**02**	**f**	**40**	**1**	**1**	**Cushing**	**A**	**GH+, PRL+, fibrous bodies, Ki 2 %**	**1**	**0**	**0**	**Sparsely granulated GH/PRL-PA**	**PA, GH sparsely granulated (0.95)**	**Loss in chr. 11**
						**B**	**ACTH+, Ki 2 %**	**0**	**1**	**0**	**Sparsely granulated ACTH-PA**	**PA, ACTH (0.99)**	**Gain in chr. 6**
**03**	**f**	**57**	**1**	**1**	**Cushing**	**A**	**ACTH++, 1 %**	**0**	**1**	**0**	**Sparsely granulated ACTH-PA**	**PA, ACTH (0.99)**	**Gain in chr. 1**
						**B**	**FSH+, LH+, ** **Ki 1 %**	**0**	**0**	**1**	**FSH/LH-PA**	**PA, FSH/LF (0.99)**	**No sign. alterations**
04	m	31	0	0	Acromegaly	A	GH+++, PRL+, TSH+, fibrous bodies, Ki 5 %	1	0	0	Sparsely granulated GH/PRL-PA	Lack of DNA	n.a.
						B	no hormones, Ki 15 %	0	0	0	Atypical null-cell-PA	Lack of DNA	n.a.
05	m	55	0	0	Acromegaly	A	GH+++, PRL+, Ki < 1 %	1	0	0	Densely granulated bihormonal bicellular GH/PRL-PA	Lack of DNA	n.a.
						B	LH+, Ki < 1 %	0	0	1	LH-PA	Lack of DNA	n.a.
06	m	45	1	0	Acromegaly	A	GH+++, PRL++, α-sub++, LH+, fibrous bodies, Ki 2 %	1	0	0	Sparsely granulated GH/PRL-PA	Lack of DNA	n.a.
						B	FSH+, Ki 2 %	0	0	1	FSH-PA	PA FSH LH (0.99)	No sign. alterations
07	f	45	0	0	Cushing	A	ACTH+++, Ki 5 %	0	1	0	Densely granulated ACTH-PA	Lack of DNA	n.a.
						B	FSH+, LH+, Ki 2 %	0	0	1	FSH-LH-PA	Lack of DNA	n.a.
08	f	54	0	0	Acromegaly	A	GH+++, PRL+, fibrous bodies, Ki < 3 %	1	0	0	Sparsely granulated GH/PRL-PA	Lack of DNA	n.a.
						B	ACTH+++, Ki < 3 %	0	1	0	Densely granulated ACTH-PA	Lack of DNA	n.a.
**09**	**m**	**55**	**0**	**0**	**Cushing**, **Hyperprolac** **-tinaemia**	**A**	**PRL+++, Ki 1–2 %**	**1**	**0**	**0**	**Densely granulated PRL-PA**	**PA, GH densely granulated (0.71)**	**Loss in chr. 2, 13, 18, 19; gain in chr. 3, 5, 7, 8, 9, 12, 14**
						**B**	**ACTH+++, ** **Ki 1–2 %**	**0**	**1**	**0**	**Densely granulated ACTH-PA**	**PA, ACTH (0.88)**	**Loss in chr. 6, 10, 11, 17, 19**
10	m	59	0	0	Acromegaly	A	GH++, Ki < 1 %	1	0	0	Densely granulated GH-PA	Lack of DNA	n.a.
						B	LH+, Ki < 1 %	0	0	1	LH-PA	Lack of DNA	n.a.
**11**	**f**	**65**	**1**	**1**	**Acromegaly**	**A**	**GH+++, PRL+, fibrous bodies, Ki 1 %**	**1**	**0**	**0**	**Sparsely granulated GH-PA**	**PA, GH densely granulated group B (0.99)**	**Gain in chr. 5**
						**B**	**No hormones, Ki < 1 %**	**0**	**0**	**0**	**Null-cell-PA**	**PA, TSH (0.88)**	**Gain in chr. 1, loss in chr. 1, 11, 18, 22**
12	m	81	1	1	Hyperprolac -tinaemia	A	PRL+++, Ki n.a.	1	n.a.	0	Densely granulated PRL-PA	Lack of DNA	n.a.
						B	FSH +, LH +, Ki n.a.	0	n.a.	1	FSH/LH-PA	Lack of DNA	n.a.

Bold face: Cases depicted in the *t*-SNE plot (Fig. 3) and chromosomal gains and losses (Fig. 4)

*N.a.* not available, *m* male, *f* female, *0* absent, *1* present, age in years, + < 20% positive cells, ++ 20% to < 60% positive cells, +++ ≥ 60% positive cells, *Ki* percentage of Ki67 positive nuclei, *n. d.* not determined, *PA, FSH LH* gonadotroph pituitary adenoma, *PA ACTH* adrenocorticotroph hormone producing pituitary adenoma, *PA TSH* thyrotroph hormone producing pituitary adenoma, *PA GH DNS B* densely granulated somatotroph hormone producing pituitary adenoma group B, *PA GH SPA* sparsely granulated somatotroph hormone producing pituitary adenoma

All cases were re-classified according to the current WHO Classification of Tumours of Endocrine Organs [13] by three experienced pathologists/neuropathologists (WS, RB, CH). The immunoreactivity of the hormones and keratin was assessed as percentage of slightly, medium, or strongly positive cells. The values were simplified into three grades: less than 20% positive cells: slight staining (+), 20% to less than 60% positive cells: medium staining (++), 60% and more positive cells: strong staining (+++). Ki67 was evaluated as percentage of positive nuclei. Nuclear expression of transcription factors was rated as negative or positive (0/1).

### DNA methylation profiling and copy number analyses

In 4 cases, the paraffin blocks contained enough clearly separated tissue of the two PAs for DNA methylation profiling. For DNA isolation from FFPE tissue, ten 10 µm sections were cut and DNA was isolated using the ReliaPrep™ FFPE gDNA Miniprep System (Promega) according to manufacturer’s instructions. About 100–500 ng DNA was used for bisulfite conversion by the EZ DNA Methylation Kit (Zymo Research). Afterwards, the DNA Clean & Concentrator-5 (Zymo Research) and the Infinium HD FFPE DNA Restore Kit (Illumina) were employed to clean and restore the converted DNA. Finally, either the HumanMethylation450 BeadChip array or the Infinium Methylation EPIC BeadChip Kit (both Illumina) were used to quantify the methylation status of 450,000 or 850,000 CpG sites, respectively, on an iScan device (Illumina). Read outs of the arrays were processed according to a previously published brain tumour classification [14]. Copy number profiles were generated using the ‘ChAMP’ package for “R” environment (http://bioconductor.org/packages/release/bioc/html/ChAMP.html, last accessed October 12, 2020).

## Results

An overview of the results is depicted in Table 1.

### Clinical data

The cohort comprised 6 female and 6 male patients (mean age 55.4 yrs., ranging from 31 to 81 years, Table 1). All patients presented with endocrine symptoms, six with acromegaly, four with Cushing’s disease, one with hyperprolactinaemia, and one with Cushing disease and hyperprolactinaemia. Upon radio-imaging, two tumours of acromegalic patients (cases 6, 11), two lesions of patients with Cushing’s disease (cases 2, 3), and one tumour of a woman with hyperprolactinaemia (case 12) appeared as macro adenoma (diameter 1–4 cm). All of these also showed an invasive growth into neighbouring structures except for one patient with acromegaly (case 6).

### Histopathology and immunohistochemistry

**Fig. 1:**
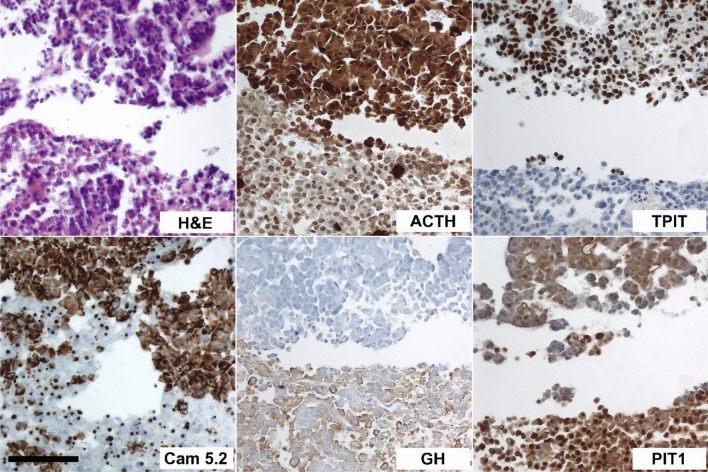
Double adenoma comprising a sparsely granulated ACTH adenoma and sparsely granulated GH/PRL adenoma (case 2). Two clearly separated fractions of solid growing epithelial tumors. The upper tumour part comprises cells with faintly basophilic cytoplasm (H&E) and cytoplasmic expression of ACTH and cytokeratin (Cam5.2) as well as nuclear expression of TPIT. The lower tumour fraction in contrast, shows small cytoplasmic cytokeratin positive inclusions (fibrous bodies), faint cytoplasmic expression of growth hormone (GH) and PIT1 positive nuclei. Scale bar lower left = 100 μm, applies for all pictures

**Fig. 2:**
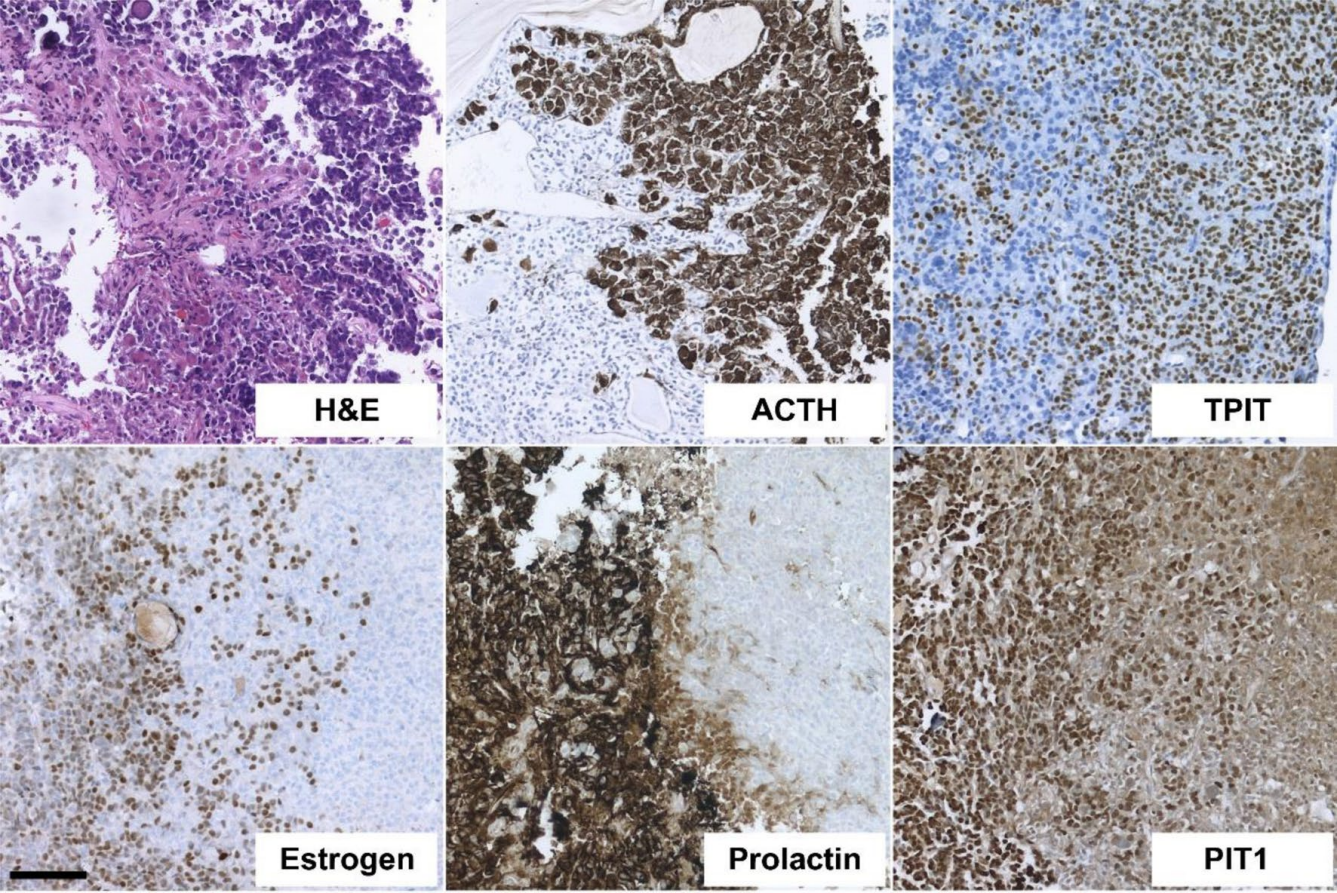
Double adenoma composed of a densely granulated ACTH adenoma and a densely granulated prolactin adenoma (case 9). Two partly intermingled epithelial tumours consisting of a solid, slightly fibrotic PA composed of small eosinophilic cells (H&E) with nuclear expression of estrogen receptor and PIT1 as well as strong cytoplasmic expression of prolactin (left side of the photographs), and a fraction of slightly larger, basophilic cells (H&E) with nuclear expression of TPIT and strong cytoplasmic positivity for ACTH (right side of the photograph). Scale bar lower left = 100 μm, applies for all pictures

All cases were recognized as double pituitary adenoma (DPA) of diverse cell lineages according to their cytology and hormone expression pattern. Investigation of tissue samples revealed clearly separated tumour entities with fragments of diverse cellular differentiation (see Fig. 1), but also DPA with a contiguous growth and partly admixed cells of diverse hormone or transcription factor expression (see Fig. 2). Among the 12 DPA investigated, the most frequently associated tumour types comprised GH/PRL/TSH and FSH/LH DPAs (cases 5, 6, 10, 12) followed by GH/PRL/TSH adenoma combined with an ACTH adenoma (cases 2, 8, 9) and corticotroph adenoma combined with a FSH/LH lesion (cases 3, 7). Two GH/PRL/TSH adenoma were associated with a null-cell adenoma (cases 4, 11), and in one case (case 1, ACTH-FSH/LH adenoma) the transcription factor SF1 was not detected, hence it may be argued that this was a monomorphous plurihormonal PA. A combination of corticotroph and null-cell adenoma or a FSH/LH adenoma in association with a null cell adenoma was not observed. An increased proliferative activity (Ki67 > 3%) was observed in 3 tumours of two patients (case 4: sparsely granulated GH PA, Ki67 = 5% and null-cell PA, Ki67 = 15%; case 7: densely granulated ACTH PA, Ki67 = 5%).

Invasive tumour growth which is often observed in clinically hormone inactive lesions, was present in 5 cases of our cohort, four of which comprised a gonadotroph tumour component (cases 1, 3, 12) or null cell-adenoma (case 11). Increased proliferative activity was not associated with a large tumour size or invasive growth.

### Molecular pathology

**Fig. 3:**
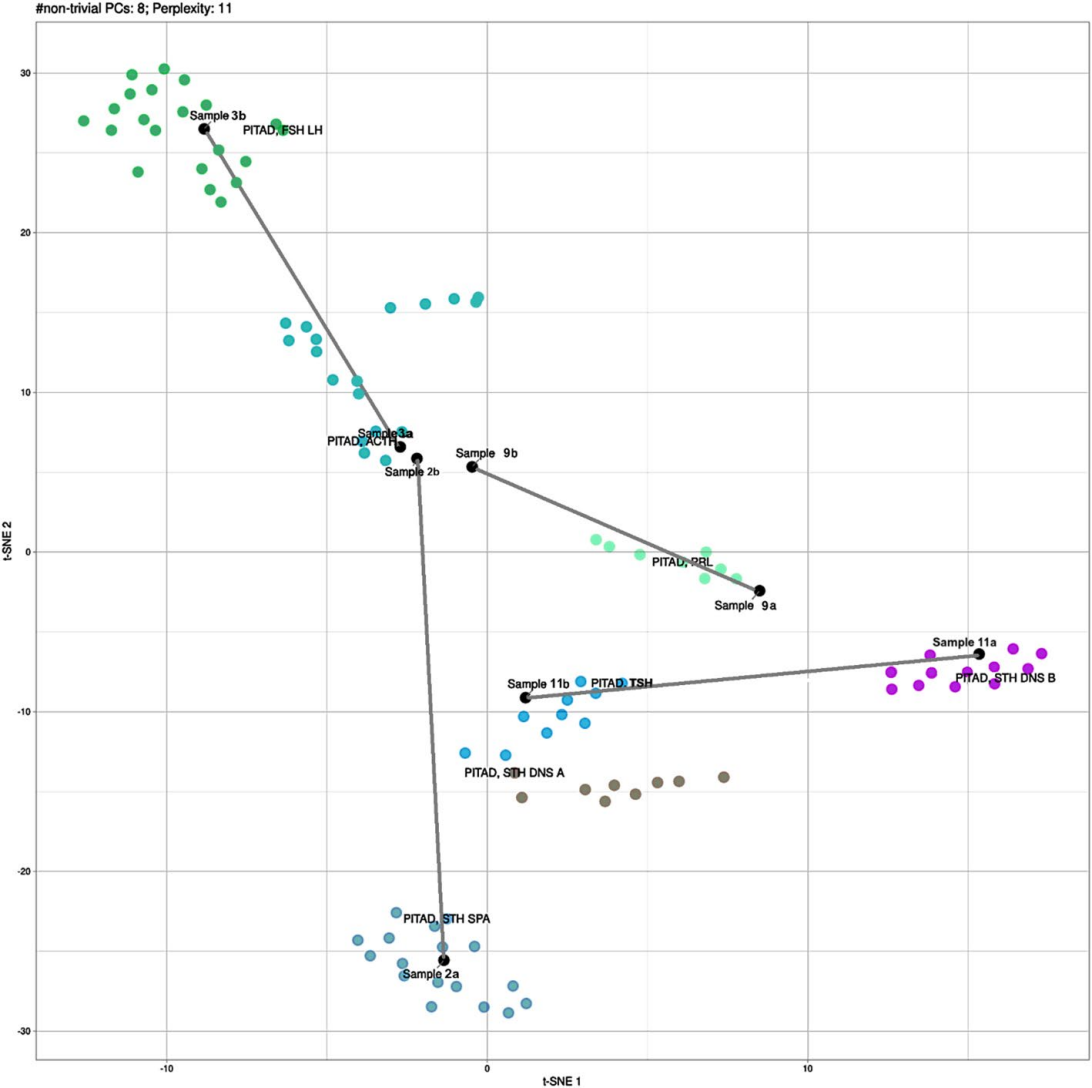
*t*-SNE of 4 pairs of double adenomas together with reference cohorts of PA subclasses. Global DNA methylation pattern of 8 samples from 4 patients were compared to reference methylation groups of PA as defined by Capper et al. 2018. Reference classes are depicted in color. New samples from this study are in black with two samples from one patient being connected by a grey line. Similarities and differences of global DNA methylation pattern are illustrated by *t*-distributed stochastic neighbor embedding (*t*-SNE). *PITAD, FSH LH* gonadotroph pituitary adenoma; *PITAD ACTH*: adrenocorticotroph hormone producing pituitary adenoma, *PITAD PRL* prolactinoma; *PITAD TSH* thyrotroph hormone producing pituitary adenoma, *PITAD GH DNS A* Densely granulated somatotroph hormone producing pituitary adenoma group A, *PITAD GH DNS B* Densely granulated somatotroph hormone producing pituitary adenoma group B, *PITAD GH SPA* Sparsely granulated somatotroph hormone producing pituitary adenoma

**Fig. 4:**
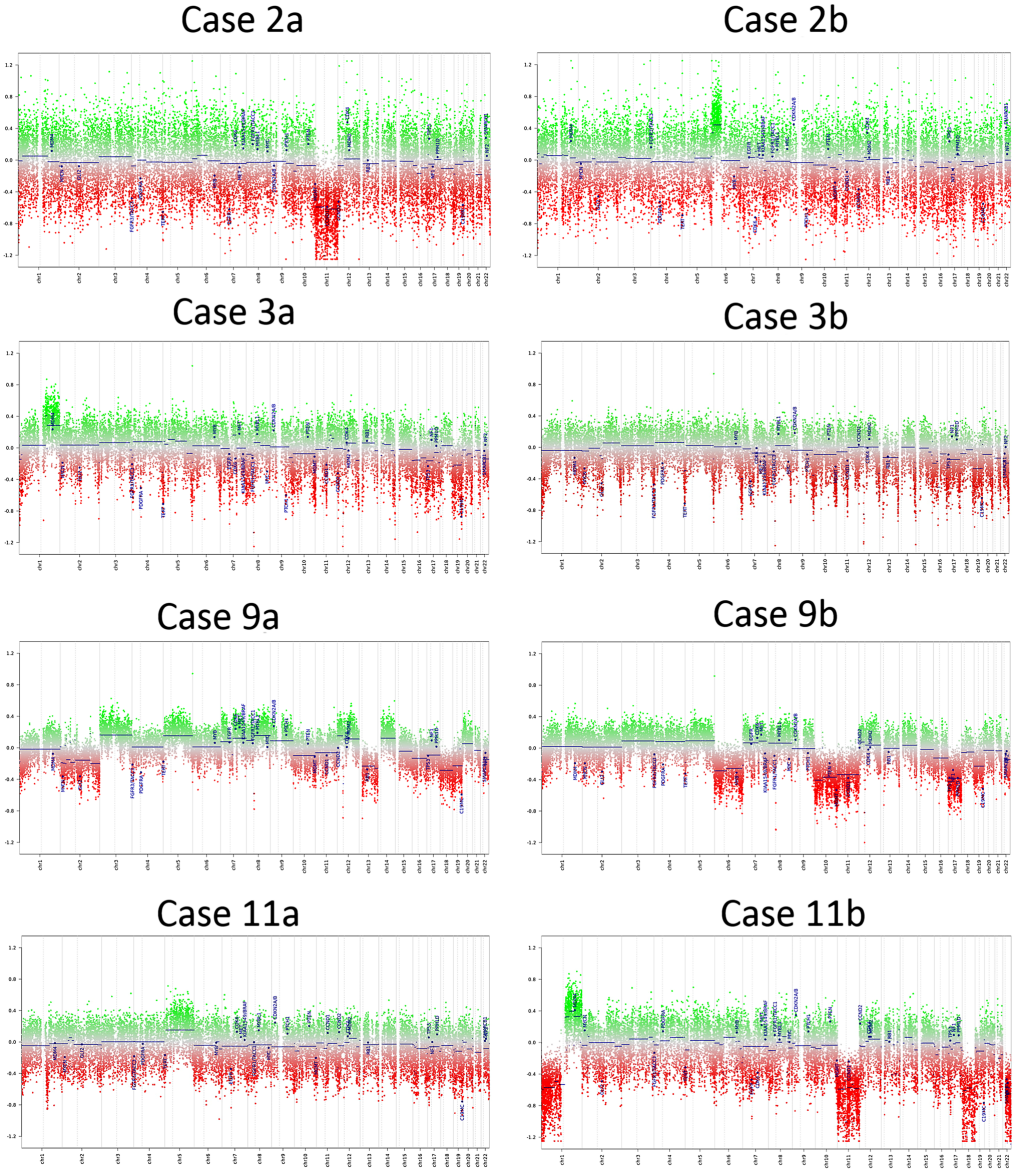
Chromosomal gains and losses in 4 pairs of double PAs. Copy number variation plots from 4 pairs of double PAs show clear differences in the two tumors of a single patient

Expression of different hormones and distinct transcription factors in each of the multiple tumours within one patient is already a strong hint pointing towards independent neoplasms that occur simultaneously, but origin from precursor cells of different cell lineages. In order to confirm these findings on a genetic and epigenetic level, we microdissected specimens from 8 tumours originating from 4 patients and performed global DNA methylation profiling. Using a previously published algorithm that was established to classify brain tumours based on DNA methylation [14], we realized that in all 4 patients, from whom sufficient tumour material was available, the two tumours showed distinct DNA methylation profiles matching to two different tumour methylation classes (Table 1). Illustrating the proximity of these tumours to each other and to previously published reference classes by *t*-distributed neighbor embedding (*t*-SNE) again confirmed the epigenetic differences of different tumours occurring in one single patient (Fig. 3). Looking at copy number variations, for which the data can be inferred from the DNA methylation data, we finally demonstrated that differences between two tumours in one patients are already visible on a genetic level. As shown in Fig. 4, each of the analysed tumours revealed chromosomal gains or losses that were absent from its counterpart in the same patient. For example, the sparsely granulated GH cell adenoma in case 2 demonstrated a loss of chromosome 11, whereas the ACTH cell adenoma in the same patient showed gains on chromosome 6p (Fig. 4).

## Discussion

In the present study, the hormone and transcription factor expression was assessed in a series of 12 double pituitary adenomas (DPA) and compared with results of DNA methylation profiling and chromosomal copy number analyses in 4 cases.

Age (range 31 to 81 years, mean 55.4 years) and gender (6 female and 6 male patients) of the 12 DPA patients roughly conformed to epidemiological data reported for monomorphous PA [15]. However, children and adolescents were not part of our cohort, which may suggest that DPA develop successively, presenting clinically at a slightly higher age than monomorphous PA. If this was true, it may further be speculated that a clinically silent or unrecognized PA develops first, followed by a symptomatic tumour, which then leads to diagnosis. Concerning the frequency, DPA were estimated to account for 0.5–1.5% of surgical PA specimens [4, 5]. The lower percentage of 0.3% in our cohort (12 cases out of 3654 (+6 hypophyseal carcinomas) supposedly resulted from our selection of cases as we had to rely on diverse hormone expression pattern for identification of DPA and thus missed cases with diverse expression of transcription factors which were not assessed before 2017. Further, adenomas arising as separate tumours of the same cell lineage are not recognized histologically as different lesions.

The DPA demonstrated 4 lineage combinations: SF1-PIT1 (n = 4), SF1-TPIT (n = 3), TPIT-PIT1 (n = 3), and PIT1-null cell (n = 2). In accordance with the literature, the combinations of GH/prolactin/TSH—FSH/LH adenoma (n = 4), GH/prolactin/TSH – ACTH (n = 3), and ACTH–FSH/LH adenoma (n = 3) were most commonly observed [16]. Different expression pattern of hormones were confirmed by different expression of transcription factors in 11/12 patients.

Concerning the 8 tumours that were additionally analysed by global DNA methylation profiling the histomorphological and immunohistochemical classification was confirmed in most cases. However, in one case (case 11b) that was immunohistochemically classified as null-cell adenoma global DNA methylation profiling revealed a TSH PA. Here, the PIT1 antibody reacted with the adjacent GH PA, but did not recognize the TSH PA. The differential clustering of PA of the same lineage in the *t*-SNE plots may thus indicate further differences in the adenoma types as mentioned in the introduction.

Still, our data on DPA demonstrate that a complete and precise microscopic diagnostic approach is indispensable for the diagnostics of pituitary tumours and renders a valid diagnosis in most cases. However, DNA methylation profiling and copy number analyses may be extremely useful if the microscopic picture is not clear. This is true for all PA, but particularly valuable, if a DPA appears to be present.

In conclusion double pituitary adenomas are readily diagnosed by histomorphological and immunohistochemical evaluation in most cases. Global DNA methylation profiling may yield additional information in lesions that appear as null-cell adenomas immunohistochemically.

## Data Availability

Data are available from the corresponding author upon request.
